# Towards elucidating the radiochemistry of astatine – Behavior in chloroform

**DOI:** 10.1038/s41598-019-52365-5

**Published:** 2019-11-04

**Authors:** Emma Aneheim, Stig Palm, Holger Jensen, Christian Ekberg, Per Albertsson, Sture Lindegren

**Affiliations:** 1 Department of Radiation Physics, Institute of Clinical Sciences, Sahlgrenska Academy,University of Gothenburg, SE41345 Gothenburg, Sweden; 2 Department of Oncology, Institute of Clinical Sciences, Sahlgrenska Academy, University of Gothenburg, SE41345 Gothenburg, Sweden; 3 Region Västra Götaland, Sahlgrenska University Hospital, Department of Oncology, SE41345 Gothenburg, Sweden; 4 Department of Energy and Materials – Nuclear Chemistry, Institute of Chemistry and Chemical Engineering, Chalmers University of Technology, SE41296 Gothenburg, Sweden; 5 PET and Cyclotron Unit, Copenhagen University Hospital, KF3982 Copenhagen, Denmark

**Keywords:** Organic chemistry, Nuclear chemistry, Inorganic chemistry

## Abstract

Targeted alpha therapy of disseminated cancer is an emerging technique where astatine-211 is one of the most promising candidate nuclides. Although astatine has been known for over 70 years, its chemistry is still largely unexplored, mainly due to the lack of stable or long-lived isotopes. However, substantial amounts of astatine-211 can be produced in cyclotrons by the bombardment of natural bismuth. The astatine can be recovered from the resulting irradiated target material through either wet extraction or dry-distillation. Chloroform has become an important intermediate solvent for the recovery of astatine after production, especially following dry distillation. In this work, the radiochemistry of astatine in chloroform was investigated using evaporation, solvent extraction, chromatographic methods and molecular modeling. The extraction of astatine in chloroform led to the formation of multiple astatine species, allowing for evaporation of the solvent to dryness without any loss of activity. Radiolysis products of chloroform were shown to play an important role in the speciation of astatine forming both reactive and kinetically stable compounds. It was hypothesized that reactions with chlorine, as well as trichloromethyl hydroperoxide, forming polar astatine compounds are important reactions under the current experimental conditions.

## Introduction

Astatine was first synthesized at UC Berkeley in 1940 by Corson, MacKenzie, and Segrè^1^. This method involves the bombardment of natural bismuth-209 with medium energy alpha particles in a cyclotron^1–3^. This method can be used to produce both astatine-210 and 211, which are the astatine isotopes with the longest half-lives of 8.1 and 7.2 hours, respectively. Astatine isotopes 214–219 are, however, found in the earth’s crust, in equilibrium with uranium, but it has been estimated that as little as 0.07 gram exists at any given time^4,5^, which is why astatine is often called “the rarest element on earth”. Thus, little is known about the properties of astatine and its behavior is based on its position in the halogen group in the periodic table^6^. Some of the basic properties of astatine can be extrapolated from its neighboring halogen, iodine. However, the chemistry of astatine differs significantly from that of iodine, which can be partially explained by the higher relativistic contribution in chemical bonding and hence the greater metallic nature of the element^7,8^. Another factor influencing its behavior in chemical systems is that all the astatine isotopes apart from three (astatine-221, 222 and 223), are alpha particle emitters^5^. Radiolysis induced by alpha particles can hence play a significant role in the behavior of astatine in many chemical systems compared to that of stable or β-emitting isotopes of iodine, due to the nature and yield of the radiolysis products formed^9^.

Astatine-211 is of particular interest for use in targeted alpha therapy for disseminated cancer. Alpha particles have a short range in tissue (<100 µm) and high energy, resulting in both a high linear energy transfer and relative biological effectiveness^10,11^. Only a few alpha particle emitting nuclides, such as astatine-211, bismuth-213, thorium-227, radium-223, actinium-225 and lead-212/bismuth-212, fulfill most of the physical requirements for applications in nuclear medicine^12–14^. Among these astatine-211 is one of the most promising candidates due to its physical properties and simple production route^15^. However, the chemistry of astatine is significantly less established than that of other alpha emitters, due to the lack of long-lived or stable isotopes^16^ although significant advances have been made in basic astatine chemistry in the past decade^8,17–19^. Astatine-211 has 100% alpha emission with only one alpha particle per decay. This characteristic prevents unpredictable localization of the dose caused by the detachment of radioactive daughters from the carrier vector, as is the case with thorium-227, radium-223, lead-212/bismuth-212 and actinium-225, which all have long decay series or suffer from recoil problems^5,15,20,21^. Although cell internalization may prevent the daughter activity from escaping from the cell, this factor places very high demands on the delivery method^21^. On the other hand, the alpha decay of astatine requires higher activities to be administered to the patient than with serially decaying radionuclides to achieve the same cytotoxicity. The half-life of astatine-211 is 7.2 h, which is long enough to allow for efficient labeling chemistry and compatibility with a number of different carrier vectors, compared, for example, to the half-life of bismuth-213, which is 46 minutes^5^. It should, however, be noted that the known astatine-carbon bonds are relatively unstable *in vivo*, currently limiting systemic use of the nuclide^22^. The carrier vector can be, *e.g*. a small molecule, a peptide or a monoclonal antibody^23^, which carries the radioactivity to the site of the tumor. Astatine-211 is today predominately produced in cyclotrons using the original method developed by MacKenzie, and Segrè by the following reaction ^209^Bi (α, 2n)^211^At. The use of astatine for cancer therapy was first proposed in the early 1950s^24^ and astatine-211 has since been used in numerous pre-clinical^14,25–30^ and clinical studies^31–33^.

Few research labs around the world work with astatine because its short half-life requires relatively close proximity to a medium energy cyclotron capable of producing astatine^2^. In addition to the production, a chemical infrastructure to retrieve astatine from the irradiated target material is required^34^. Astatine recovery from irradiated target material can be performed with good yields either through dry-distillation or wet extraction^2,34–37^. Both methods involve treatment of astatine using organic diluents such as chloroform or methanol. Speciation of astatine in such organic diluents is difficult and the state of the nuclide is generally not known^38^. One aspect, which is difficult to consider in basic chemical investigations, is the dense ionization of the media surrounding the short-lived alpha emitters. This factor is particularly important for understanding the chemistry relevant to nuclear medicine applications where high activities and high activity concentrations of the alpha particle emitting astatine-211 are used.

Upon recovery of astatine-211 after dry distillation, astatine can be dissolved in chloroform and this solution evaporated to dryness, after which astatine can be transformed into chemically useful forms and further incorporated into organic molecules or biomolecules using various halogen binding chemistry strategies^2,39^. Thus, chloroform has become an important intermediate solvent for recovery of astatine and the subsequent labeling chemistry. However, the chemistry of astatine in this particular solvent is not well known, and this study was undertaken to shed light on this.

## Results and Discussion

### Evaporation studies

Astatine-211 dissolved in chloroform directly from elution after target recovery using dry distillation was found to be completely retained (101.2 ± 0.2%) during evaporation to dryness (six repetitions of 62 ± 9 MBq, 1.43 MBq/µL). This chloroform solution containing astatine after dry distillation is designated herein as “Chloroform Eluate”. Comparatively, if small activities of ^131^I dissolved in chloroform were evaporated to dryness, almost half of the activity (48 ± 5%) was released from the solvent. This result highlights the difference in chemistry between astatine and iodine, but could also be a result of the large difference in dose delivered to the solvent during the two experiments.

**Figure 1:**
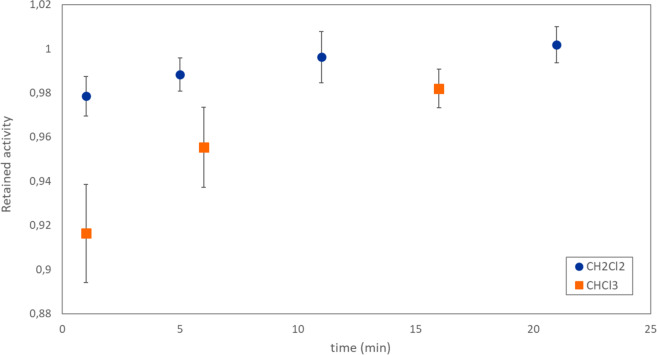
Portion of retained activity after evaporation of redissolved evaporated Chloroform Eluate as a function of time after redissolution in chloroform (CHCl_3_) and dichloromethane (CH_2_Cl_2_).

To compare astatine behavior in other solvents with the results in chloroform, portions of the Chloroform Eluate were evaporated to dryness and then redissolved in fresh solvent, before subsequent evaporation of the new solvent. When this process was performed using chloroform as the redissolution solvent, a clear relationship was observed between time after redissolution and remaining activity after subsequent evaporation (Fig. 1). It should be noted that in this experiment, dose delivered to the bulk solvent was significantly lower than in the former direct evaporation of Chloroform Eluate, and can explain why the astatine speciation may differ. However, as the activity concentration in the solvent rapidly increases during the course of an evaporation experiment the astatine speciation is likely to change during the course of the procedure and the final species may be the same both for Chloroform Eluate and redissolved Chloroform Eluate. A similar behavior was observed when changing the redissolution diluent to dichloromethane (Fig. 1). However, the use of methanol resulted in no time dependent behavior and a significantly smaller portion of retained activity (51 ± 4%). In a previous report, the stabilization of astatine using N-chloro succinimide was necessary for subsequent reactions with organic tin molecules after astatine recovery from distillation using methanol^39^. This outcome indicate that the presence of another halogen is important for the formation of stable astatine species, allowing for evaporation to dryness. In addition, the time-dependence seen in evaporation studies suggests that the formation of stable astatine species also is dependent on the absorbed dose to the solvent.

Direct labeling of protein-based targeting molecules starting with astatine in chloroform requires evaporation of the solvent. Upon redissolution in an aqueous medium, the astatine is often reacted with a reagent such as N-iodo succinimide or chloramine T^40,41^. Thus, the astatine speciation after evaporation changes before labeling, circumventing plausible problems in labeling with difference in astatine speciation caused by dilution or change in evaporation solvent. Indirect, or two-step, labeling of proteins with astatine in chloroform has previously been shown to be problematic as a result of competing reactions with chlorine radicals formed by radiolysis of the solvent^42^.

### Solvent extraction

**Figure 2:**
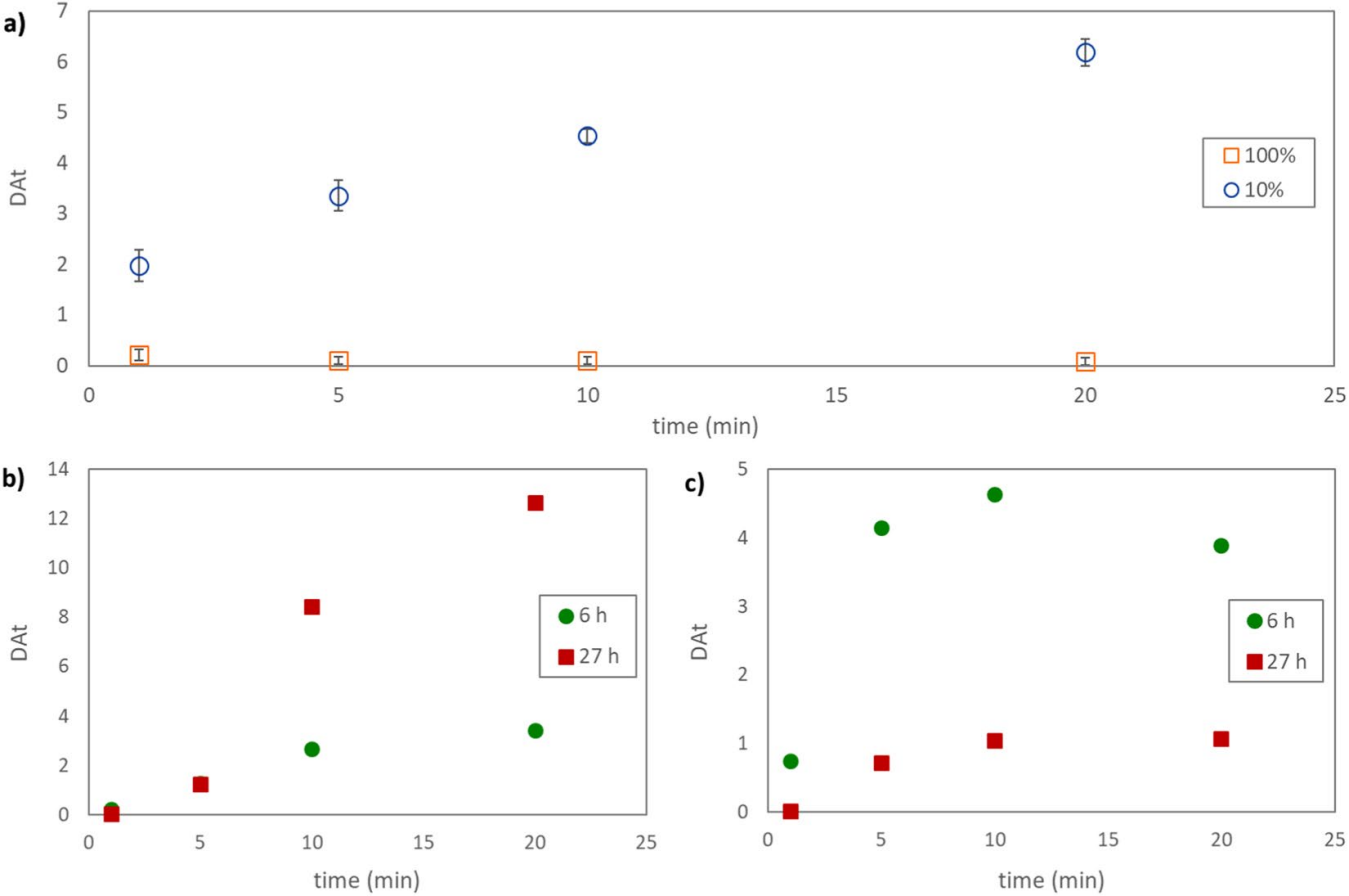
(**a**) A comparison of astatine distribution ratios (D_At_) as a function of time between Chloroform Eluate (100%) and Chloroform Eluate (10%) diluted into fresh chloroform. (**b**) D_At_ as a function of time, diluting Chloroform Eluate (10%) into fresh chloroform at different time points after elution. (**c**) D_At_ as a function of time, diluting Chloroform Eluate (10%) in fresh chloroform at different time points after elution, allowing the organic phase to react >1 h before solvent extraction experiments. In all cases the aqueous phase was 0.1 M NaClO_4_.

To investigate the speciation of astatine in Chloroform Eluate, solvent extraction experiments were carried out. Initially three different aqueous phases (0.1 M KNO_3_, 0.1 M NaClO_4_ and water) were used for extraction with Chloroform Eluate as organic phase resulting in very similar low distribution ratios of astatine activity after 10 and 20 minutes with all aqueous phases (D_At_ = 0.018 ± 0.008). Thus, less than 2% of the astatine activity remained in the organic phase after contact with the different aqueous phases. These values are similar to previously found astatine distribution ratios from similar aqueous phases into chloroform^43^. When comparing solvent extraction experiments performed using 0.1 M NaClO_4_ as the aqueous phase and Chloroform Eluate as organic phase with a dilution of 10% Chloroform Eluate in fresh chloroform, the results differed significantly, both regarding extraction kinetics and equilibrium distribution ratio (not reached for the diluted series) (Fig. 2, panel a). For the investigated time-points, the low astatine distribution ratio for Chloroform Eluate was independent of contact time while for the diluted Chloroform Eluate the astatine distribution ratio increased, reaching a value of 6.2 ± 0.3 after 20 minutes of contact. This result indicate that astatine speciation rapidly changes with the introduction of fresh chloroform, forming extractable compounds. It was postulated previously that astatine changes oxidation state at the phase boundary during solvent extraction experiments into chloroform through reactions with solvent components or degradation products^43^. A change in astatine speciation at the phase boundary may also be true for the current experiments where astatine is present in the organic phase before the start of the solvent extraction experiments. The chemical form(s) of astatine in these experiments are hence difficult to determine. The finding, however, correlate well with the evaporation investigation where redissolved evaporated Chloroform Eluate behaved differently compared to directly evaporated Chloroform Eluate.

If diluted Chloroform Eluate ought to be used without additives or evaporation in the subsequent chemistry, *e.g*. in labeling of organic molecules or indirect labeling of protein-based vectors, the found complex behavior and difference in astatine speciation compared to Chloroform Eluate alone may compromise the resulting radiochemical yield.

The radioactive dose to the solvent is an important factor affecting astatine speciation, which is evident when repeating the experiments, diluting the Chloroform Eluate at later time-points after elution (Fig. 2, panel b). Waiting 27 hours after elution compared to 6 hours, before diluting the Chloroform Eluate to 10% resulted in significantly higher astatine distribution ratios and a break in the kinetic curve, indicating a change in speciation and/or extraction mechanism during the experiment. However, it was not possible to make a correlation between equilibrium distribution ratio and absorbed dose to the solvent. Solvent extraction directly with Chloroform Eluate was not dependent on time in the same way, as may be expected since in the case the solvent has already received a very high dose shortly after elution, and the results presented in Fig. 2, panel a, represent an average of 10 experiments at different time points after elution.

If the solutions diluted 6 and 27 hours after elution were allowed to react for >1 hour before performing the solvent extraction experiment, kinetics and equilibrium distribution ratio again changed (Fig. 2, panel c). For the 27 h time point, distribution ratios dropped significantly, indicating that upon dilution into fresh chloroform after receiving a high dose, extractable but reactive species were formed. A long time of contact (80 minutes) was performed for the freshly diluted samples at 6 and 27 hours after elution and, in both cases, the distribution ratio dropped. In the 27 h study the equilibrium distribution ratio approached the ratios found in Fig. 2, panel c (D_At_(6 h) = 2.8 D_At_(27 h) = 1.3). This result further strengthen the proposal of formation of reactive extractable astatine species when the Chloroform Eluate samples were diluted in fresh chloroform. It is possible that one or more of the astatine species in Chloroform Eluate is present in chemical equilibria with radiolysis products of chloroform and neat chloroform and that this equilibrium is shifted upon dilution with fresh chloroform without the presence of degradation products. The addition of fresh chloroform may also introduce additional oxygen to the reaction mixture, which can result in (additional) formation of oxygen-containing astatine species.

### Chromatographic investigations

To obtain more information about the astatine species present in Chloroform Eluate a variety of chromatographic methods were applied.

TLC experiments showed that two or more species of astatine with different polarities likely were present simultaneously in Chloroform Eluate (Table 1). Applying acetonitrile as the mobile phase resulted in separation of at least two astatine species between the solvent front and the starting application point. Applying solvent mixtures intended for the separation of polar species (dichloromethane/methanol 90:10 and ethyl acetate/methanol/acetic acid/water (80:10:5:5)) resulted in mobility of greater portions of activity compared to acetonitrile alone but with less distinct separation of components, suggesting that one or more of the present astatine species are of polar nature. Aqueous-based mobile phases resulted in significant losses of activity from the TLC plate and in many cases large portions of the activity was volatilized, which is why these results are not included in Table 1.

**Table 1:** Activity distribution on TLC strips after application of Chloroform Eluate using different mobile phases.

Mobile phase	Start	Middle	Front	Liquid
Hexane	99,6%	0,3%	0,1%	0,0%
Dichloro methane (DCM)	99,1%	0,9%	0,0%	0,0%
Methanol (MeOH)	93,4%	3,5%	2,6%	0,5%
Ethyl acetate (EtAc)	71,0%	16,7%	12,3%	0,0%
Ethanol (95%)	68,0%	15,8%	15,0%	1,2%
Acetonitrile	67,0%	4,3%	28,7%	0,0%
DCM/MeOH (90:10)	55,0%	30,2%	14,8%	0,7%
EtAc/MeOH/acetic acid/water (80:10:5:5)	19,5%	21,7%	57,0%	1,9%

In addition to TLC the behavior of Chloroform Eluate on a polar organic matrix in the form of a low pressure pre-packed column with hydrophilic styrene divinylbenzene polymer was also investigated.

Chloroform Eluate was strongly retained on the polymer resulting in <4% loss of activity when washed with water or 5% methanol in water. Removal of the activity proved difficult as <25%, and often significantly less, of the activity was eluted when applying different organic mobile phases (methanol, ethanol, acetonitrile, dichloromethane, chloroform). When comparing the use of N-chloro-succinimide (NCS) with N-bromo-succinimide (NBS) and N-iodo succinimide (NIS) in methanol/1% acetic acid as eluent it led to the conclusion that NCS removed <9% of the activity whereas NBS and NIS removed >25% of the activity. This outcome indicated that the reaction between existing astatine species and bromine as well as iodine is preferred over the reaction with chlorine, further strengthening the theory that chlorine is present in one or more of the astatine species in this setting.

To confirm the simultaneous presence of several different astatine species, as indicated by TLC, Chloroform Eluate was analyzed using a specific HPLC setup with activity detection and directly adjacent quantification^44^. HPLC analysis had previously shown the simultaneous presence of different astatine species in solution under other experimental conditions^32^.

**Figure 3:**
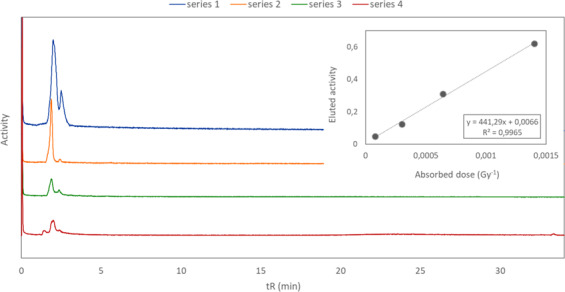
HPLC chromatograms with activity detection of Chloroform Eluate with low activity concentration (series 1–3) and high activity concentration (series 4). Absorbed dose to solvent increases from series 1–4. Insert graph show the amount of eluted activity as a function of absorbed dose to the solvent for the four series.

Analysis of Chloroform Eluate with a comparatively low activity concentration (*circa* 0.26 MBq/µl at time of elution) and injection shortly after elution  showed the presence of two different astatine species, using a gradient with high organic content (Fig. 3, series 1). However, the total area of the peaks only corresponded to 62% of the activity applied on the column, suggesting that, at least, one other astatine species, retained on the column, may be present. Repeated analysis of the same solution (series 2 and 3) as well as analysis of Chloroform Eluate with higher activity concentration (*circa* 1.0 MBq/µl at time of elution, series 4) showed that the peak area of the detected species is proportionately reversed to the absorbed dose to the solvent (Fig. 3, insert graph). A high dose to Chloroform Eluate hence results in the formation of astatine species that are completely retained on a C18 reversed phase column, despite efforts to change gradients and mobile phases. Basic compounds are known to react with the silica support and/or metal impurities in the support of HPLC columns^45^, which could indicate that the astatine species retained on the column display basic properties.

Chloroform Eluate diluted in fresh chloroform, previously shown to have a behavior different from that of only Chloroform Eluate, was also analyzed with HPLC. Similar to the case with Chloroform Eluate two early peaks were detected (Supplementary Figure S1) but when the gradient was changed, several different activity peaks with relatively long retention time were also found. A dynamic relationship between astatine speciation and absorbed dose was also evident as the relation between peak area as well as the number of peaks and their retention times shifted over time (Supplementary Figure S2). However, the combined area of the peaks in all cases correspond to less than 42% of the injected activity, which indicated that a large portion of the activity was absorbed onto the C18 column, similar to the results with directly injected Chloroform Eluate. A very low abundance of peaks with similar long  retention times could also be found when analyzing Chloroform Eluate with a low activity concentration, shortly after elution (Supplementary Figure S3).

HPLC analyses of both Chloroform Eluate and diluted Chloroform Eluate after solvent extraction were also performed (Supplementary Figure S4 and S5). The results differ from that of Chloroform Eluate and diluted Chloroform Eluate using the same HPLC gradient, indicating that astatine speciation changed upon aqueous phase contact, as could be expected.

### Molecular modeling

Speciation of astatine has generally been attempted in aqueous media at ultra-trace concentrations i.e. astatine activity concentrations <1 kBq/µl, in order to avoid the effect of radiolysis^8,18^. This can be compared to this work where activity concentrations in Chloroform Eluate generally were more than a thousand fold higher. In this work, radiolysis has also shown to  play an important role in the astatine speciation, which is why molecular modeling of astatine interactions with both neat solvent and different plausible radiolysis products of chloroform was performed to elucidate the observed experimental behavior of astatine.

Molecular modeling suggested that no direct favorable interactions take place between astatine and neat chloroform (Supplementary Table 1).

γ-Radiolysis of chloroform give rise to a number of different radiolysis products depending on dose, presence of oxygen, moisture and impurities in the solvent^9^. Besides the presence of low-molecular species such as chlorine gas and hydrochloric acid, different forms of dimers of the two diluents are commonly found. Also oxygen-containing species such as peroxides and phosgene can be found when oxygen is present during irradiation, which was the case in the experiments in this work.

**Figure 4:**
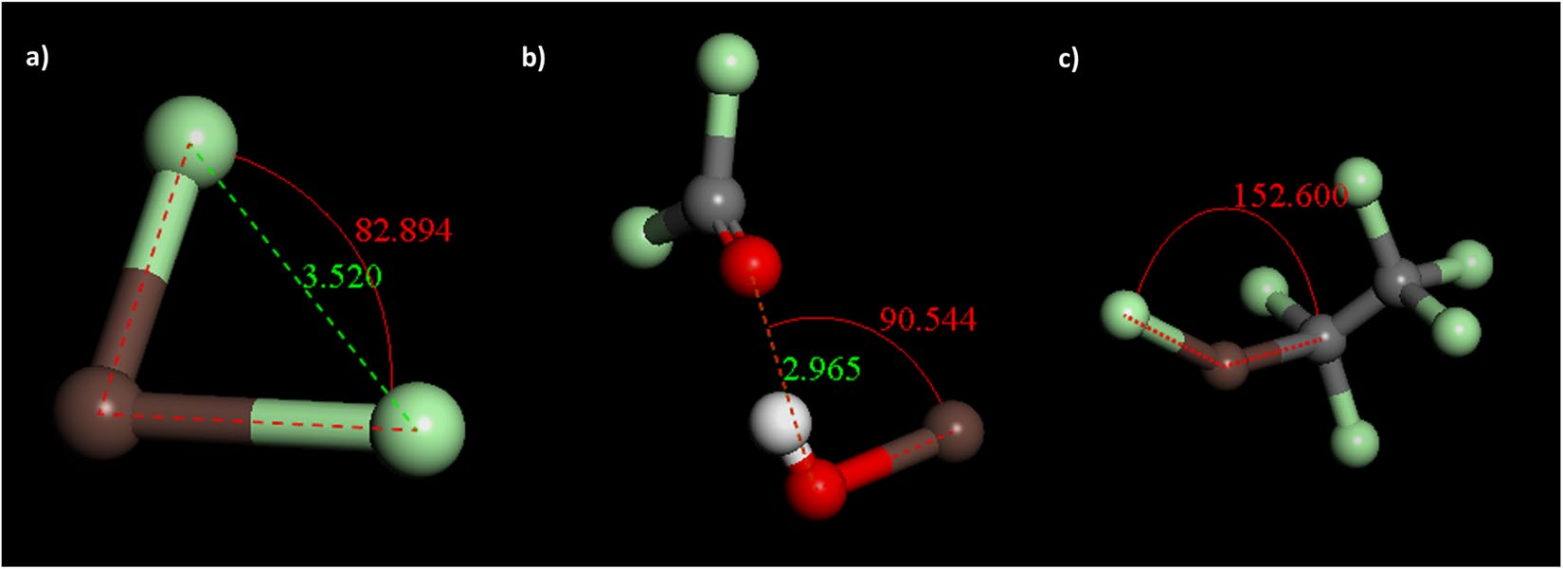
Geometry optimized structures of interactions between astatine (brown) and (**a**) Cl_2_ (**b**) CCl_2_OOH and (**c**) C_2_Cl_6_ modeled in Materials Studio 6.0, Accelrys.

Modeling suggested that energetically favorable reactions could take place with chlorine, trichloromethyl hydroperoxide or hexachloro ethane (Fig. 4, Supplementary Table 1). Repeating solvent extraction experiments both with Chloroform Eluate and 10% Chloroform Eluate diluted in fresh chloroform with the addition of hexachloroethane to the solvent did not significantly alter the resulting astatine distribution ratio. This result suggests that hexachloroethane is not the predominant radiolysis product reacting with astatine in chloroform. Under oxygenated conditions trichloromethyl hydroperoxide is the dominant radiolysis product of chloroform, which upon prolonged irradiation forms phosgene, with which no favorable interactions with astatine were found through modeling. Favorable interactions with the peroxide were found, which partly could explain the difference in behavior when diluting Chloroform Eluate with fresh chloroform as formed peroxides rapidly react further to form carbonyl chloride and hypochlorous acid, or in the presence of moisture to yield hydrochloric acid, carbon dioxide and oxygen^9^. No interaction between hydrochloric acid and astatine were found. Peroxide reactions are, however, not likely to be the only interaction taking place, as peroxides also are formed upon irradiation of methanol^9^ and the astatine evaporation behavior in methanol in this work was found to be significantly different from that in chloroform. Therefore, the interaction found with chlorine is likely to be one of the most important processes in explaining the stable behavior of astatine in chloroform upon evaporation. Radiolysis of dichloromethane also form a number of radiolysis products where chlorine atoms are intermediate products^46^, providing an explanation to the similar evaporation stability found in this solvent. Di-halogen and di-halogen binary astatine compounds such as AtI/AtBr and AtI_2_^−^/AtBr_2_^−^ as well as a ternary halogen compound (IAtBr^−^) have previously been shown to exist in aqueous solution^47^. Halogen compounds involving two or more astatine atoms are not likely to form in solution due to the low concentration of astatine atoms. The di-halogen compound between astatine and iodine, AtI, has also been shown to form halogen bonds with organic Lewis bases where astatine acts as the halogen bond donor^48^. It is therefore possible that additional reactions take place including the postulated binary di-halogen formed between astatine and chlorine, but this remain to be investigated.

## Conclusions

Astatine in chloroform forms species that are stable, allowing for evaporation of the solvent to dryness without any loss of astatine activity. Different sets of experiments suggest that multiple astatine species are present simultaneously in chloroform and that these species most likely result from interactions with chlorine and oxygen-containing radiolysis products of chloroform. The formation and nature of these species have been shown to be heavily dependent on dose to the solvent and modeling suggest that reactions with chlorine and trichloromethyl hydroperoxide are of importance. Astatine speciation has been shown to rapidly change when introducing fresh chloroform to a solution that already has received a high astatine radiation dose, which complicates radiochemical analysis of the original solution.

More extensive studies are needed to fully elucidate the radiochemistry of astatine in chloroform and other organic halogenated solvents. The effect of oxygen must be investigated by performing the experiments under inert atmosphere conditions. It is also possible that other radiolysis products and especially in different yields are formed during the described present situation with a dense α-radiation field compared to literature data that are based on γ-irradiation. In addition, the possibility that astatine forms halogen bonds in the resulting complicated organic matrixes after radiolysis of halogenated solvents should be investigated.

## Methods and Material

All aqueous solutions were prepared using ultrapure water, MilliQ (>18.2 MΩ*cm) and all organic reagents used were of analytical grade or higher.

### Astatine production and recovery

Astatine-211 was produced in the MC32 Scanditronix cyclotron at Copenhagen University Hospital by 28 MeV alpha particle bombardment of a target consisting of an aluminum backing (0.5 × 27 × 30 mm) with a bismuth layer of ∼20 µm, covered with a thin aluminum layer (∼5 µm, to hinder astatine evaporation during production). Upon arrival in Gothenburg the astatine containing (*circa* 600–800 MBq) bismuth layer was removed from the backing together with the covering aluminum layer by a mechanical scraper. The resulting powder was dry-distilled (710 °C, circa 30 s) and recovered in chloroform (300–500 µl) using an automatic system^28^. Decay corrected recovery yields were typically between 75–85% resulting in a general astatine-211 activity concentration of 1–2 MBq/µL of chloroform. The recovered astatine was then either used as-is or portioned into different fractions and evaporated to dryness.

### Evaporation

Astatine-211 recovered in chloroform after dry-distillation, termed throughout this study as Chloroform Eluate, was evaporated by placing a portion of the chloroform in a Screw Top Tapered Vial (1.1 mL) under a gentle stream of nitrogen (160–220 mL/h) directed into the vial until dryness. This process, depending on liquid volume took 0.5–5 minutes. Activity was measured before and after evaporation using a dose calibrator (VEENSTRA instruments).

Dry portions of Chloroform Eluate in Screw Top Tapered Vials (1.1 mL) were also redissolved in 200 µL of chloroform, dichloromethane or methanol. After different periods of time small portions (3 × 20 µL) from these solutions were removed and once again subjected to evaporation as described above.

Evaporation of iodine-131 containing chloroform was performed as stated above after creating the radioactive solution from H_2_SO_4_ (500 µL, 3 M) spiked with ^131^I (50 µL), subsequently oxidized with the addition of H_2_O_2_ (70 µL) under a layer of chloroform (500 µL), which then was removed from the aqueous phase.

### Solvent extraction

Organic phases were in all cases astatine containing chloroform, either recovered directly from dry distillation of astatine, Chloroform Eluate, or fresh solvent spiked with a small portion of Chloroform Eluate (10%). Unless otherwise stated, aqueous phases were either NaClO_4_ (0.1 M), KNO_3_ (0.1 M) or water and both phases were always of equal volume (40–200 µl). The organic and aqueous phases were contacted using an IKA VIBRAX mechanical vortex shaker (2000 rpm) in glass vials with screw top lid (either 2 ml or 300 µL). Equal volume samples (2–10 µL) were withdrawn from each phase for activity distribution analysis. Sampling was performed at different time-points after the beginning of contact for kinetics analysis.

Results are discussed in terms of astatine distribution ratios, determined by measuring the activity in both phases, according to the following equation:

$${D}_{At}=\frac{{[At]}_{org}}{{[At]}_{aq}}=\frac{{A}_{At,org}}{{A}_{At,aq}}$$

### Thin Layer Chromatography (TLC)

Chloroform Eluate (2–5 µL) was spotted on TLC plates with aluminum backing (Silica Gel 60 F_254_, Merck). After drying, the plates were placed in conical screw cap poly propylene tubes (50 mL) with 4 ml mobile phase (acetonitrile, hexane, methanol, ethanol (95%), dichloromethane, ethyl acetate, dichloromethane/methanol (90:10), ethyl acetate/methanol/acetic acid/water (80:10:5:5), water/ethanol (80:20), water, NaClO_4_ (0.1 M), citrate buffer (pH 5.5), NaOH (0.1 M)). When the front of the mobile phase had travelled close to the end of the plate, the plate was removed from the tube, dried, and cut in three equal parts; start (spot), middle and front, before the activity was measured in a dose calibrator.

### Low-pressure chromatography

Chloroform Eluate (5–10 µL) was added to 1 ml Bond Elut PLEXA columns (Agilent) both dry and pre-equilibrated using methanol (5%) in water. After application of the activity the columns were measured with a dose calibrator before being washed with methanol (5%) in water or water (1 mL). After washing the activity of the column was measured before elution with different types of organic solvents and solvent mixtures (1 mL). After elution, the activity of the column was measured again.

### HPLC

HPLC analyses were performed using a Jasco LC4000 Prep-LC (UV detector UV-4075, autosampler AS-4050, pump PU-4086 Binary, LC-NetII/ADC and ChromNAV software), in combination with a flow-through sodium iodide NaI(Tl) well detector used for activity analysis and quantification^44^. Analytical chemistry was conducted on a reversed-phase C18 column (YMC-Triart). After studying several different gradient systems as well as combinations of mobile phases (water, buffer of pH 6.5, methanol and acetonitrile) the following mobile phase conditions were used if nothing else is  stated: 20% A (water/ 0.1% trifluoroacetic acid (TFA)) and 80% B (acetonitrile), for 10 minutes, linear gradient to 0% A and 100% B during 5 minutes, followed by 10 minutes hold, thereafter a linear gradient to 95% A and 5% B during 5 minutes and hold for another 5 minutes.

### Molecular modeling

Density functional theory (DFT) calculations were performed using Materials Studio 6.0 from Accelrys. Gas-phase properties (energy, geometry) were determined for different combinations of astatine and chloroform as well as radiolysis products of chloroform. Calculations were made using the Generalized Gradient Approximation PW91 Functional, implementing a chloroform solvation model.

## Supplementary information

Supplementary Material

## Data Availability

All relevant data generated or analyzed during this study are included in this published article (and its Supplementary Information files).
