# Supplementary material for: Towards elucidating the radiochemistry of astatine – Behavior in chloroform
Source: Sci Rep. 2019 Nov 4;9:15900. doi: 10.1038/s41598-019-52365-5 (PMC6828679; doi:10.1038/s41598-019-52365-5)
Supplement: Supplementary file 1 — Supplementary Material [file 41598_2019_52365_MOESM1_ESM.pdf]

Supplementary Material to:

Towards elucidating the radiochemistry of astatine – Behavior in chloroform

Emma Aneheim, Stig Palm, Holger Jensen, Christian Ekberg, Per Albertsson, Sture Lindegren

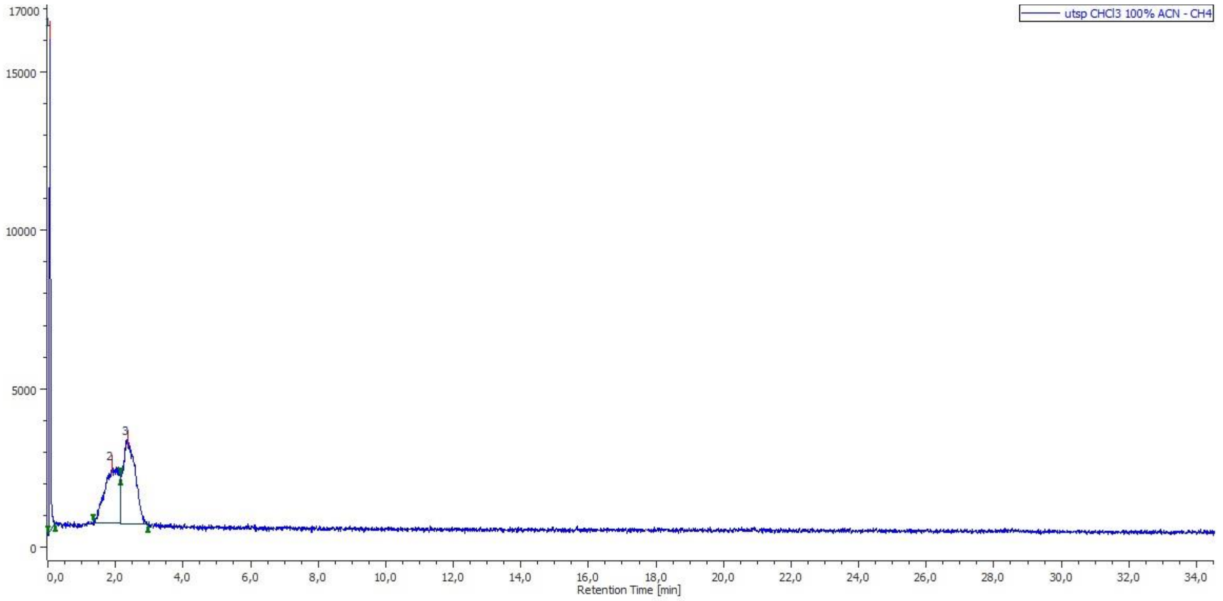

Figure S1. HPLC chromatogram with activity detection of Chloroform Eluate (10%) diluted with fresh chloroform before analysis. Gradient: 20% A (water/ 0.1% trifluoroacetic acid (TFA)) and 80% B (acetonitrile), for 10 minutes, linear gradient to 0% A and 100% B during 5 minutes, followed by 10 minutes hold, thereafter a linear gradient to 95% A and 5% B during 5 minutes and hold for another 5 minutes.

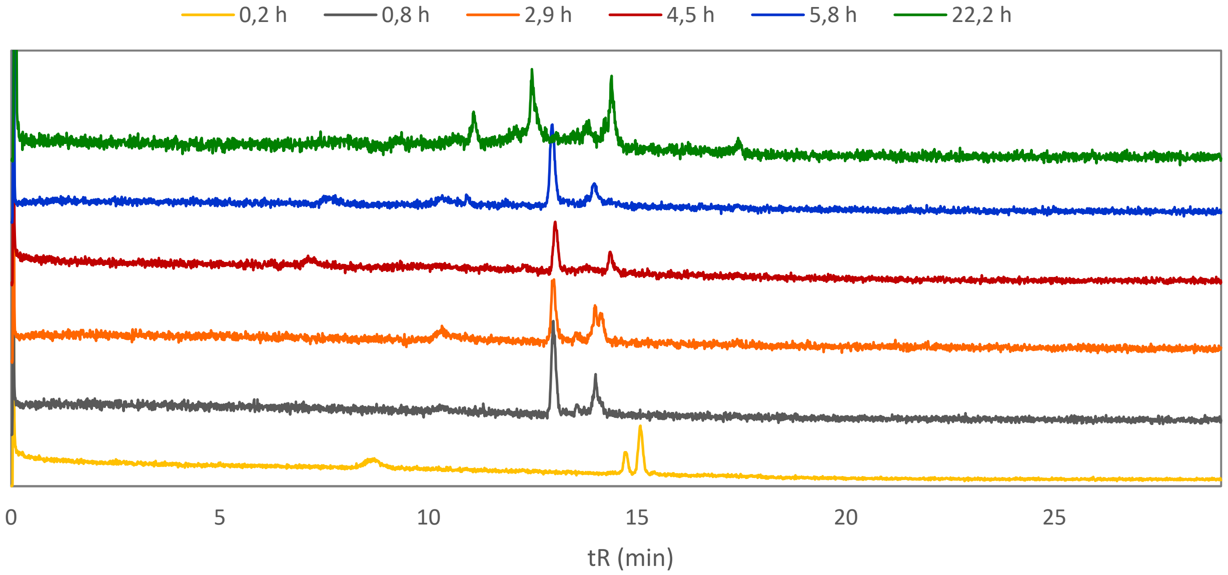

Figure S2. Combined HPLC-chromatograms (Activity detection) of Chloroform Eluate diluted in fresh chloroform (1%) at different times after dilution. Gradient: 95 % A (water with 0.1 % trifluoroacetic acid (TFA)) and 5% B (acetonitrile with 0.1% TFA), for 5 minutes, linear gradient from 95 % A and 5% B to 5 % A and 95% B during 10 minutes, followed by five minutes hold, thereafter a linear gradient back to starting conditions during 5 minutes and hold for another 5 minutes.

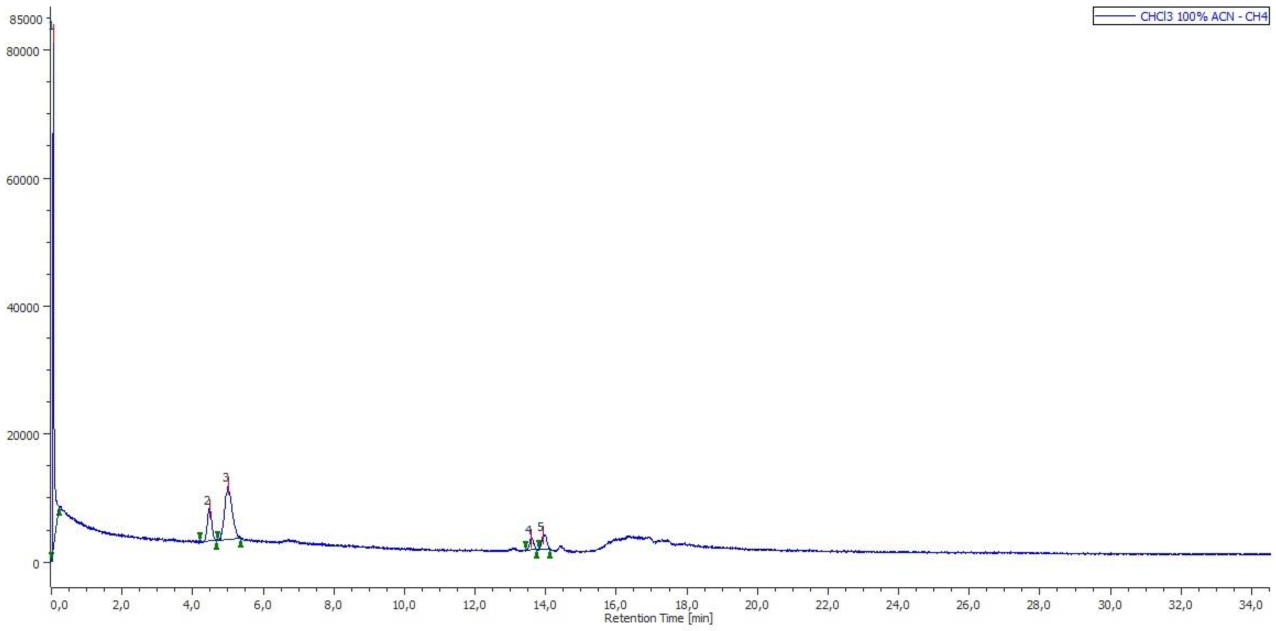

Figure S3. HPLC chromatogram with activity detection of Chloroform Eluate (low activity concentration 0,26 MBq/ul, injection circa 30 minutes after elution). Gradient: 30% A (water/ 0.1% trifluoroacetic acid (TFA)) and 70% B (acetonitrile), for 5 minutes, linear gradient to 0% A and 100% B during 10 minutes, followed by 5 minutes hold, thereafter a linear gradient to 80% A and 20% B during 5 minutes and hold for another 5 minutes

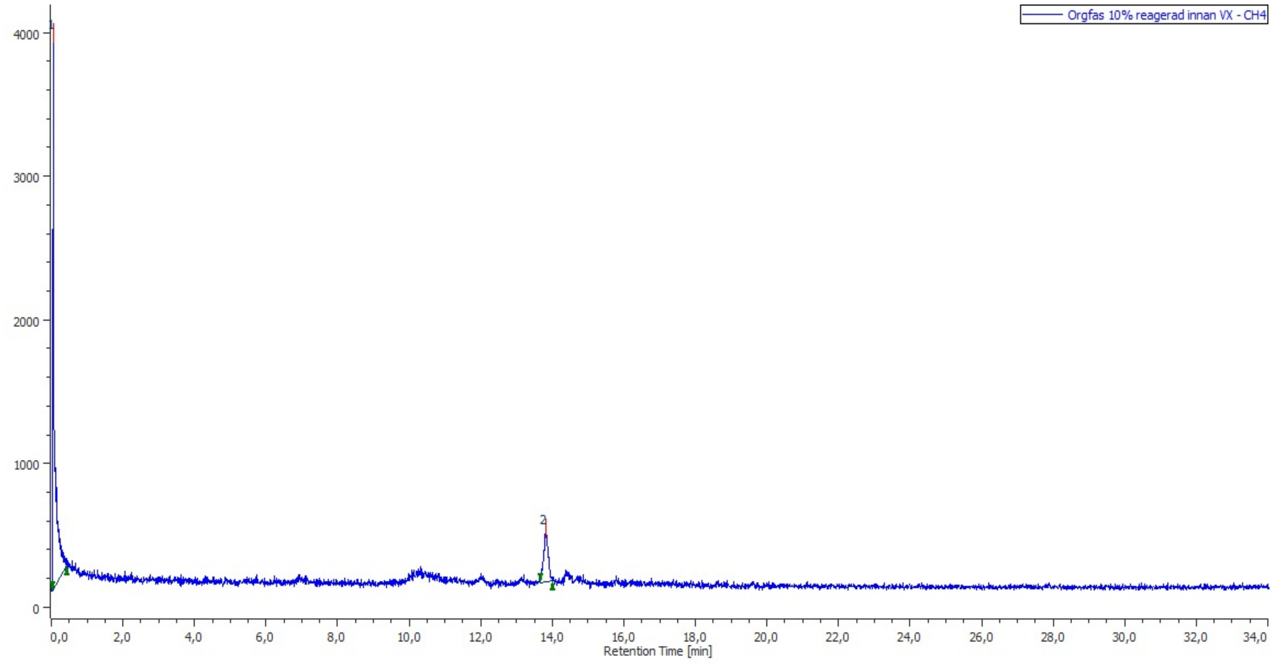

Figure S4. HPLC chromatogram with activity detection of Chloroform Eluate after contacting with an aqueous phase for 20 minutes. Gradient: 95 % A (water with 0.1 % trifluoroacetic acid (TFA)) and 5% B (acetonitrile with 0.1% TFA), for 5 minutes, linear gradient from 95 % A and 5% B to 5 % A and 95% B during 10 minutes, followed by five minutes hold, thereafter a linear gradient back to starting conditions during 5 minutes and hold for another 5 minutes.

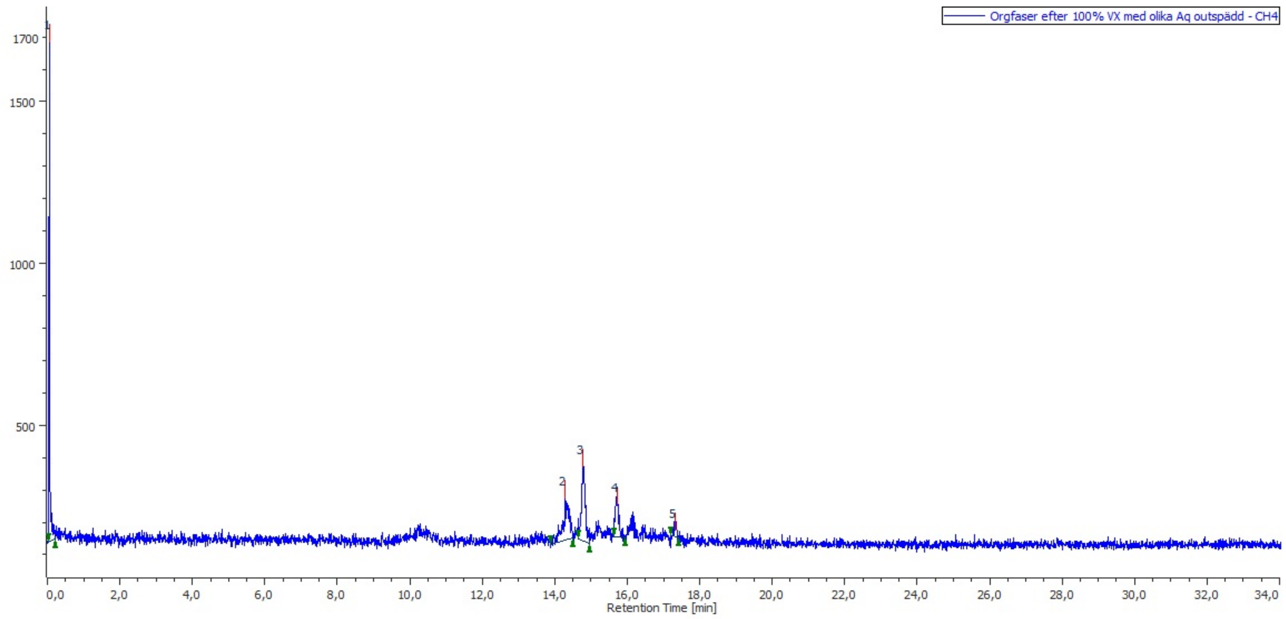

Figure S5. HPLC chromatograms with activity detection of Chloroform Eluate (10%) diluted with fresh chloforom before contacting with an aqueous phase for 20 minutes. Gradient: 95 % A (water with 0.1 % trifluoroacetic acid (TFA)) and 5% B (acetonitrile with 0.1% TFA), for 5 minutes, linear gradient from 95 % A and 5% B to 5 % A and 95% B during 10 minutes, followed by five minutes hold, thereafter a linear gradient back to starting conditions during 5 minutes and hold for another 5 minutes.

Table S1 - Astatine interactions with chloroform, chlorine hydrochloric acid (HCl) tri-chloro methyl-peroxide (peroxide), fosgene and hexachloroethane (Hexa) modelled using Materials Studio 6.0, comparing single species with combined species.

**At specie**	**Compound**	**Solvation model**	**Energy diff. (Ha)**	**Energy diff. (kJ/mol)**
At(0)	Chloroform	-	0.00906	23.78703
At(0)	Chloroform x 3	-	0.011522	30.25101
At(0)	Chloroform (CCl3At + Cl)	CHCl3	0.017517	45.99088
At2	Chloroform (2CCl3At + Cl2)	CHCl3	0.052999	139.1489
At(0)	Cl2	CHCl3	-0.023004	-60.397
At2	Cl2	CHCl3	-0.004448	-11.6782
At2	Cl2 (2AtCl)	CHCl3	-0.016245	-42.6512
At2	HCl	CHCl3	0.020635	54.17719
At2	HCl (2AtCl + H2)	-	0.05200	136.526
At2	HCl (2AtCl + H2)	CHCl3	0.055994	147.0122
At0	Peroxide	CHCl3	-0.095434	-250.562
At0	Chloroform + peroxide	CHCl3	-0.100416	-263.642
At2	Peroxide	CHCl3	-0.042187	-110.762
At2	Peroxide (alt. Config.)	CHCl3	-0.01395	-36.6257
At0	Fosgene	-	0.004204	11.0376
At0	Chloroform + Fosgene	CHCl3	0.015903	41.75333
At2	Hexa (C2Cl4At2 + Cl2)	CHCl3	0.057151	150.05
At2	Hexa (C2Cl6At2)	CHCl3	0.009463	24.84511
At0	Hexa (C2Cl5At-Cl)	CHCl3	-0.008221	-21.5842
